# Recent Progress in Cryopreservation of Bovine Oocytes

**DOI:** 10.1155/2014/570647

**Published:** 2014-03-16

**Authors:** In-Sul Hwang, Shinichi Hochi

**Affiliations:** ^1^Interdisciplinary Graduate School of Science and Technology, Shinshu University, Tokida 3-15-1, Ueda, Nagano 386-8567, Japan; ^2^Animal Biotechnology Division, National Institute of Animal Science, Rural Development Administration, Seosuwon-ro 143-13, Suwon, Gyeonggi-do 441-706, Republic of Korea; ^3^Faculty of Textile Science and Technology, Shinshu University, Tokida 3-15-1, Ueda, Nagano 386-8567, Japan

## Abstract

Principle of oocyte cryoinjury is first overviewed and then research history of cryopreservation using bovine oocytes is summarized for the last two decades with a few special references to recent progresses. Various types of cryodevices have been developed to accelerate the cooling rate and applied to the oocytes from large domestic species enriched with cytoplasmic lipid droplets. Two recent approaches include the qualitative improvement of IVM oocytes prior to the vitrification and the short-term recovery culture of vitrified-warmed oocytes prior to the subsequent IVF. Supplementation of L-carnitine to IVM medium of bovine oocytes has been reported to reduce the amount of cytoplasmic lipid droplets and improve the cryotolerance of the oocytes, but it is still controversial whether the positive effect of L-carnitine is reproducible. Incidence of multiple aster formation, a possible cause for low developmental potential of vitrified-warmed bovine oocytes, was inhibited by a short-term culture of the postwarm oocytes in the presence of Rho-associated coiled-coil kinase (ROCK) inhibitor. Use of an antioxidant *α*-tocopherol, instead of the ROCK inhibitor, also supported the revivability of the postwarm bovine oocytes. Further improvements of the vitrification procedure, combined with pre- and postvitrification chemical treatment, would overcome the high sensitivity of bovine oocytes to cryopreservation.

## 1. Introduction

Many reproductive biotechnologies have been applied to efficient production of large domestic animals, such as pigs and cattle with the high economic importance. In cattle, those originally developed in the decades of 1950s to 1970s include artificial insemination and multiple ovulations/embryo transfer combined with or without cryopreservation of spermatozoa [1] and preimplantation-stage embryos [2, 3], respectively. Successful cryopreservation of spermatozoa and embryos made these technologies more practical and available for commercial use, because of their potential advantages to allow long-distance transportation and to omit estrous synchronization, thus reducing the number of recipient female population to be maintained. Thereafter, embryo production by in vitro maturation (IVM) and in vitro fertilization (IVF) using immature oocytes from abattoir-derived ovaries and frozen-thawed spermatozoa became more or less routine since the decade of 1980s [2, 3], and production of cloned embryos has been promising with the progress of somatic cell nuclear transplantation [4].

Cryopreservation of unfertilized oocytes can be combined with these advanced reproductive technologies, in addition to its potential advantage as oocyte banking for preserving female genetic resources. Revivability of cryopreserved oocytes from small rodents and humans is extremely high, adapting well to the maintenance of the huge numbers of gene-modified transgenic strains and the efficient use in therapies for human infertility [5, 6]. However, in bovine species, the low fertilization rates and developmental competence of cryopreserved oocytes still need to be improved. Some review articles regarding this topic are available in cattle [7, 8] and pigs [8, 9]. In this paper, principle of oocyte cryoinjury is first overviewed. Then, research history of cryopreservation using bovine oocytes is summarized for the period from 1992 to 2013 with a few special references to very recent progress.

## 2. Basic Cryobiology for Oocytes

### 2.1. Events around Fertilization

Once oocytes resume the first meiotic division, the nuclear envelop (germinal vesicle: GV) is disintegrated, allowing the nuclear material to mix into the cytoplasm. Some alterations also occur in organelles such as mitochondria, cytoskeleton, and cortical granules. Microfilaments of actin are involved in cell shape modifications and movements, and microtubules (cylindrical bundle, composed from heterodimer of *α*- and *β*-tubulin) form the spindle apparatus [10]. A spermatozoon has a pair of distinct centriolar structures as the proximal centriole located within the connecting piece under the sperm head and the distal centriole organized vertically to the proximal counterpart and aligned with the sperm tail [11]. During fertilization in most mammalian species including cattle, spermatozoal centrosome, composed from the two centrioles and the pericentriolar materials such as *γ*-tubulin, centrin, and pericentrin, is brought into an oocyte. The centrosome plays a critical role in assembly of the microtubule network (sperm aster) that brings both male and female pronuclei to the center of the newly formed zygote [12]. Thus, the centrosome is considered to be the microtubule-organizing center (MTOC), with duplication during the pronuclear stage and the subsequent separation to serve as mitotic centers anchoring the chromosomes during the first cleavage [13, 14]. Abnormalities of the spindle/MTOC function/sperm aster have been shown to directly correlate with the loss of developmental potential after IVF, because they are crucial for completion of the second meiosis, extrusion of the polar body, migration of the pronuclei, and formation of the first mitotic spindle [15].

### 2.2. High Cryosensitivity of Oocytes

Biological activity is completely stopped at very low subzero temperature, and the cell viability and functional state may be preserved for long terms [16]. However, some physical stresses can damage cells at the various subzero temperatures. Intracellular ice formation is one of the biggest causes to cell damage; hence, the freezing protocols use a combination of dehydration, freezing point depression, supercooling, and intracellular vitrification in an attempt to avoid cell damages [17]. Therefore, it is important to use cryoprotective additive (CPA), such as dimethyl sulfoxide (DMSO), ethylene glycol (EG), or glycerol alone or in combination, when cryopreserving cells in any methods. Due to both of hydrophobic and hydrophilic characteristics, as well as the relatively small molecular weight, these CPAs are permeable to the plasma membrane. On the other hand, use of CPA induces some adverse effects such as osmotic injury and toxicity of the CPAs.

Incidence of cryoinjuries depends on the size and shape of the cell, the permeability of the cell membranes, and the quality of the cells. However, these factors differ from species, developmental stage, and origin [18]. Although offspring has been born using frozen-thawed oocytes from various species, the ability to support embryo development following cryopreservation procedures is still low. This may be attributed to the susceptibility of oocytes to damage during cooling and/or freezing and subsequent thawing because of their complex structure. Unfertilized oocytes are much larger than the blastomeres of an early embryo and therefore have a small surface to volume ratio [13]. This led to dehydration and penetration of CPA being difficult to achieve, which attributes to the difficulty in cryopreservation. Furthermore, the plasma membranes of oocytes differ significantly from those of embryos, partially due to the lack of aquaporin expression which affects the movement of water and CPAs [19]. There is a rise of intracellular free calcium during fertilization, which makes the ionic strength and membrane potential of the plasma membrane [20]. Other adverse effects of cryopreservation procedures include the fracture damage in zona pellucida [21] and the destruction of intercellular coupling via gap junctions between cumulus cells and the oocyte [22, 23]. In general, the primary criteria to assess postthaw viability of oocytes are the presence or absence of membrane degeneration, cytoplasmic abnormalities and zona pellucida fractures [24]. Recent studies in humans have examined the meiotic spindle using a polarized microscope apparatus, which allows the visualization of the polymerization of the meiotic spindle after vitrification and warming. However, this technique is difficult in domestic animal species due to their high cytoplasmic lipid content, which hinders spindle examination. Therefore, such dark oocytes from domestic species must be typically examined through invasive methods, such as fluorescence microscopy and biochemical or molecular analyses [25].

**Figure 1:**
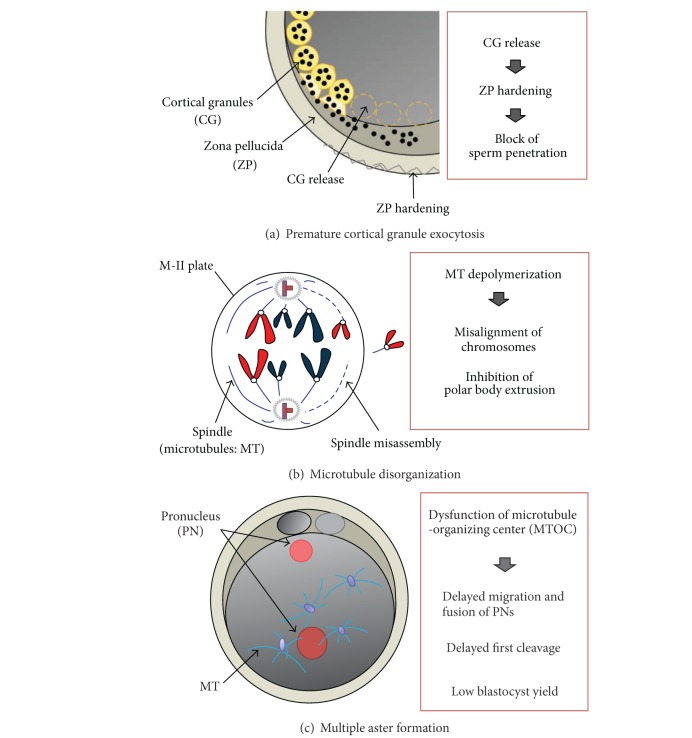
Hypotheses regarding cryoinjuries in mammalian oocytes. (a) Premature cortical granule exocytosis causes the hardening of zona pellucida, leading to block of sperm penetration. (b) Disorganization of microtubules means depolymerization of tubulin proteins, leading to misassembly of meiotic spindles, and subsequently resulting in misalignment of chromosomes and inhibition of the second polar body extrusion. (c) Multiple aster formation, resulting from ooplasmic dysfunction to support MTOC, is a possible cause of low blastocyst yield.

Low fertilization rates of cryopreserved oocytes were reported to be associated with chilling and freezing injuries, including zona hardening due to premature release of cortical granules [22, 26] and spindle disorganization and loss or clumping of microtubules [27, 28]. Briefly, exposure of mature oocytes to CPA and/or chilling procedure induced the transient rise of intracellular free calcium and prevented the sperm entry via block mechanisms at the level of plasma membrane or zona pellucida [29–32]. These processes also result in damage to the meiotic spindle, actin filaments, chromosomal dispersal, and microtubule depolymerization [33, 34]. In addition, Hara et al. [35] proposed a third hypothesis for cryodamage of bovine oocytes that multiple aster formation frequently observed in vitrified-warmed and fertilized oocytes may be related to loss of ooplasmic function responsible for normal microtubule assembly. These possible hypotheses responsible for cryodamages of the oocytes are shown in Figure 1.

## 3. History

### 3.1. Learning from Embryo Cryoresearch

Knowledge can be concurrently accumulated from research history of embryo cryopreservation. Following the first successful freezing of mouse 8-cell stage embryos in 1972 [36], pregnancy from a cryopreserved cattle embryo was reported by Wilmut and Rowson [37]. These initial findings were then extended to embryos from several mammalian species including domestic animals [38–40] and human [41]. The protocol most commonly used for successful embryo cryopreservation at that time required slow cooling from upper −7°C to below −80°C in phosphate-buffered saline supplemented with DMSO or glycerol as a permeable CPA. During the slow cooling, embryonic blastomeres are dehydrated in response to the osmotic pressure that gradually increases with the formation of extracellular ice crystals after ice seeding. The frozen embryos were warmed very slowly to avoid the rapid influx of extracellular water into the dehydrated cells during warming. This earlier protocol was labor-intensive and time-consuming.

In 1977, a two-step freezing method was reported using sheep and cattle embryos [42]. The slow cooling of embryos is interrupted at around −30°C to −36°C, followed by rapid cooling to −196°C. The embryos in LN_2_ are believed to contain intracellular ice, although it is not detrimental at this point. But to survive, the frozen embryos must be warmed rapidly to avoid injury caused by recrystallization of the intracellular ice. This two-step freezing regimen allows the development of a temperature-controlled, programmable freezer and is still used widely for many mammalian species. In cattle, pregnancy rates following transfer of embryos frozen in this way range from 50 to 60% [43]. Additional progress resulted from the use of EG as a CPA for embryos from domestic species. Using sucrose as an osmotic buffer, direct transfer of postthaw embryos into recipients without expelling them from the straws was reported by Leibo [44].

Then, a great breakthrough for simple and efficient cryopreservation has been reported by a very high cooling rate of fully dehydrated mouse embryos in highly concentrated solutes. Rall and Fahy [53] developed a novel approach to cryopreserve mouse embryos in 1985. This protocol involves dehydration of the embryos by exposing them to highly concentrated CPAs prior to cooling them to low temperature, rather than during the cooling process itself. The dehydrated embryos are rapidly cooled by being directly plunged into LN_2_. Since the cryoprotective solution can be transformed into a stable glass without ice crystal formation during the rapid cooling process, this extremely rapid method of cryopreservation is referred to as “vitrification,” meaning “glass formation.” The application of vitrification as an alternative to conventional freezing can reduce the equipment required, but technician-dependent performance of vitrification process is the limited factor for its widespread use. So far, successful vitrification producing pregnancy and/or birth of live offspring has been reported with preimplantation embryos from various mammalian species including human. A wide variety of vitrification solutions and protocols have been employed even for the same type of embryo, that is, the same species and developmental stage.

### 3.2. Cryopreservation of Mature Oocytes

Cryopreservation of oocytes has short and less successful history when compared to the other reproductive cells as spermatozoa and embryos. The first successful IVF and birth of live offspring using frozen-thawed mouse oocytes was reported in 1976 by Parkening et al. [54], and followed by Whittingham [55] and Leibo et al. [56]. Other than the mouse, such a slow freezing procedure was acceptable for species whose oocytes are not sensitive to chilling, such as cat [57, 58] and human [59]. There are a few reports regarding successful pregnancies from frozen-thawed bovine oocytes [60, 61]. However, oocytes from the large domestic species are rich in cytoplasmic lipid droplets and very sensitive to chilling, resulting in the poor revivability following the slow cooling [62]. After the publication of innovative results by Rall and Fahy [53], vitrification has been attempted to apply to oocytes. Pregnancies or birth of live offspring have been published in mouse [63], human [64], and cattle [46], with an increased requirement for improving developmental competence of the vitrified-warmed oocytes.

In 1996, Martino et al. [47] reported that 15% of matured bovine oocytes developed into blastocysts following vitrification, under in vitro culture (IVC) conditions in which >40% of the non-treated fresh oocytes were able to develop to that stage. That protocol, a pioneer work opening the new window for oocyte cryobiology, is characterized by the extremely rapid cooling rate of oocytes suspended in <1 *μ*L of a vitrification solution consisting of 30% EG plus 1.0 M sucrose placed onto electron microscope grids, a procedure derived from methods to cryopreserve Drosophila embryos [65]. The microgrids provide a cooling rate estimated to be <150,000°C/min, in contrast to 2,500°C/min with the conventionally used plastic straws. Vajta et al. [48] reported an alternative way of ultra-rapid cooling for vitrification of bovine oocytes. When the oocytes were aspirated with 20% EG and 20% DMSO solution into open-pulled straws (OPS) and cooled by directly plunging into LN_2_, 13% of the post-warm oocytes could develop into blastocysts after IVF and IVC. The OPS method has been improved to use open-pulled glass capillaries [66, 67] or commercially available gel-loading tips [68] using different CPA combinations. Other types of cryodevices so far reported for ultra-rapid cooling are the “Cryoloop” [21] and “Cryotop” [69]. Complete containerless methods have also been reported from two independent laboratories [49, 70]. Blastocyst yields from frozen-thawed or vitrified-warmed bovine metaphase-II oocytes, reported during the last two decades [45–52], are summarized in Table 1. There was no significant improvement on the blastocyst yield from cryopreserved bovine mature oocytes (commonly exceeding 10%), even after increased cleavage rates as >60% by using different cryodevices and vitrification protocols were obtained.

### 3.3. Cryopreservation of Immature Oocytes

Cryopreservation of immature oocytes at the GV stage is also the subject for challenging endeavour. Vajta et al. [48] reported that 25% of bovine oocytes vitrified-warmed using OPS system could develop into the blastocyst stage on Day 8. While it is still unclear that the high revivability of post-warm GV oocytes in the OPS system is reproducible, birth of calves following transfer of embryos derived from cryopreserved immature oocytes [71] encouraged such challenges. Abe et al. [72] reported that 8% of bovine oocytes developed into blastocysts when they were exposed to EG + Ficoll + sucrose-based solution in a stepwise manner and vitrified-warmed on nylon-mesh holder as a cryodevice, with successful data on a live calf after transfer. Bovine oocytes at the GV stage have homogenous (=less variable in size) lipid droplets that show little change following cooling, but intercellular coupling via gap junctions between cumulus cells and the GV-stage oocytes may be sensitive to osmotic stress. In addition, maintaining functional integrity of the cumulus cells after vitrification and warming is an important factor to harvest cytoplasmically-matured oocytes after subsequent IVM process.

## 4. Recent Improvement of Oocyte Cryosurvival

Our literature search failed to find recent papers published during 2011 to 2013 which described the significantly improved cryosurvival (blastocyst yield) of bovine oocytes by modifying the CPA composition, the cryodevice, or the CPA addition/dilution process in the vitrification procedures. Hence, a few chemical treatments of bovine oocytes during the IVM prior to vitrification and during the recovery culture after vitrification are highlighted in this section. Using mouse and porcine embryos, cellulose triacetate hollow fiber with a pore size of 7.5 nm has been proposed as a new device that can vitrify large amount of embryos without stepwise handlings of the embryos, reported recently by Matsunari et al. [73]. But this hallow fiber vitrification procedure has not yet been applied to the bovine oocytes.

### 4.1. Treatment During IVM

**Figure 2:**
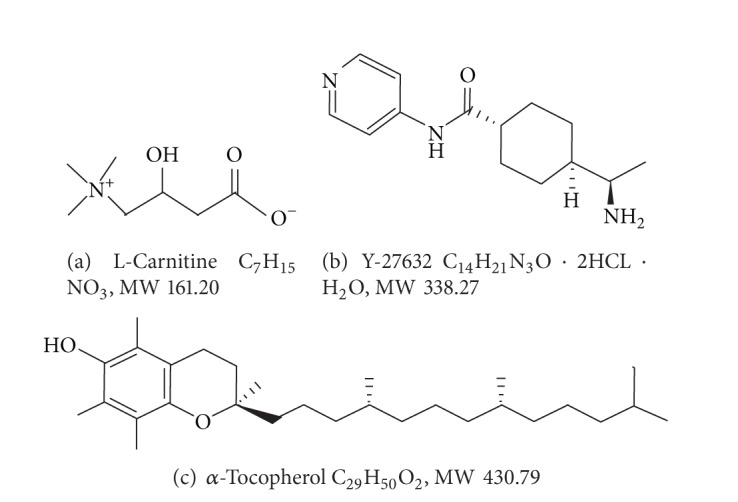
Structures of chemicals used for improvement of cryosurvival of bovine oocytes and resulted in significantly higher blastocyst yield in vitro [84, 85]. (a) L-carnitine, (b) ROCK inhibitor Y-27632, (c) *α*-tocopherol.

Large amount of cytoplasmic lipid droplets serves as energy resource but increases sensitivity of bovine oocytes to chilling injury during cryopreservation. Most of the lipid droplets locate at the periphery of plasma membrane or close proximal to organelles such as mitochondria and endoplasmic reticulum [74], both of which are the major target of cryodamage in oocyte organelles [75, 76]. Cytoplasmic lipid droplets can be partially removed from bovine oocytes by high magnitude centrifugation, and incidence of polyspermic penetration in the centrifuged and vitrified-warmed oocytes was significantly inhibited (blastocyst yield; 11% versus 7% in noncentrifuged control) [61]. It is also well known that vitrification of bovine oocytes induces mitochondrial dysfunction and loss of adenosine triphosphate (ATP) [77, 78]. L-Carnitine (Figure 2(a)), an active form of carnitine, can enhance lipid metabolism in animal cells and play an important role in the transportation of fatty acids from the cytoplasm to the mitochondria for *β*-oxidation [79]. Hence, L-carnitine can enhance ATP production in animal cells [80] and stimulate mitochondrial metabolism during maturation as firstly reported in mouse oocytes [81]. In porcine oocytes enriched with cytoplasmic lipid droplets, supplementation of L-carnitine into IVM medium reduced the amount of the lipid droplets and changed their distribution from the cortex to the medulla of oocyte cytoplasm [82]. Furthermore, supplementation of L-carnitine into IVC medium reduced the lipid content in bovine embryos and increased cryotolerance and developmental competence [83].

Two recent papers have described the effect of L-carnitine supplementation into IVM medium for bovine oocytes on their cryotolerance [84, 86], as summarized in Table 2. Both groups have employed Cryotop vitrification system for cryopreservation of bovine mature oocytes, but composition of the vitrification solution was different between the groups. Chankitisakul et al. [84] showed that bovine oocytes matured in the presence of 0.6 mg/mL; L-carnitine had the higher developmental potential to blastocyst stage 8 days after vitrification and IVF when compared to those matured in the absence of the L-carnitine (34% versus 20%, fresh control; 44%). No significant changes were found in nuclear maturation rate, ATP content, timing of first cleavage, and blastocyst quality, while dislocation of lipid droplets from the peripheral area to inner cytoplasm was observed in the L-carnitine treated oocytes. On the other hand, negative result of L-carnitine treatment during IVM on cryotorelance of bovine oocytes has been reported by Phongnimitr et al. [86]. The attempt of this research group in Thailand seemed to be conducted almost simultaneously with the above Chankitisakul's group (based on the date of initial paper submission). Supplementation of 0.6 mg/mL L-carnitine to the IVM medium significantly improved the nuclear maturation rate (78% versus 68%) and the Day-7 blastocyst yield from non-vitrified control oocytes (31% versus 24%), but did not contribute to improve the cryotolerance of the oocytes (Day-7 blastocyst yield; 13% versus 11%). Further research would be needed to clarify the effect of L-carnitine on bovine oocytes.

Glutathione (L-*γ*-glutamyl-L-cysteinyl-glycine; GSH), a major nonprotein sulfhydryl compound, plays an important role in protecting cells against the destructive effects of reactive oxygen species (ROS) and regulating syntheses of DNA and proteins [87]. GSH level increases during oocyte maturation in the ovary and reaches a peak at the metaphase-II stage [88]. However, the GSH levels of IVM oocytes are lower when compared with those of ovulated oocytes, as reported in some species [89–92]. GSH synthesis in oocytes during IVM may be disturbed by a low availability of cysteine [87, 93]. Low molecular weight thiol compounds, such as *β*-mercaptoethanol and cysteamine, can promote cysteine (cystine) uptake through formation of a mixed disulfide compound [94, 95]. In addition, such thiol compounds supplemented into IVM medium can increase intracellular GSH level and the developmental potential of the bovine oocytes [96]. It has been reported that GSH in bovine IVM-IVF oocytes can stimulate sperm aster formation [97]. Therefore, we have produced bovine IVM oocytes with 2.5-fold higher GSH content and then vitrified-warmed using Cryotop [98]. However, the high content of GSH in mature oocytes did not result in suppression of the high incidence of multiple aster formation (vitrified; 61% versus 53%, fresh control; 16% versus 17%) and improvement of developmental potential into Day-8 blastocysts (17% versus 16%, fresh control; both 41% regardless of thiol treatment for GSH).

### 4.2. Postvitrification Treatment

Generally, increased apoptosis in embryonic cells by oocyte vitrification procedure results in a decrease of developmental competence [99, 100]. Rho-associated coiled-coil kinase (ROCK), which is a kinase belonging to the AGC (PKA, PKG, and PKC) family of serine-threonine kinases, was realized as a downstream target of the small GTP-binding protein Rho [101], which could regulate growth, adhesion, migration, metabolism, and apoptosis of cells through controlling the actin-cytoskeletal assembly and contraction of cells [102]. Inhibition of the ROCK activity was involved in decrease of apoptosis in embryonic stem cell-derived neural cells [103]. Inhibition of the ROCK activity was also effective to improve the plating efficiency of dissociated human pluripotent stem cells after cryopreservation [104–108] and the revivability of in vitro produced bovine blastocysts after vitrification and warming [109].

**Figure 3:**
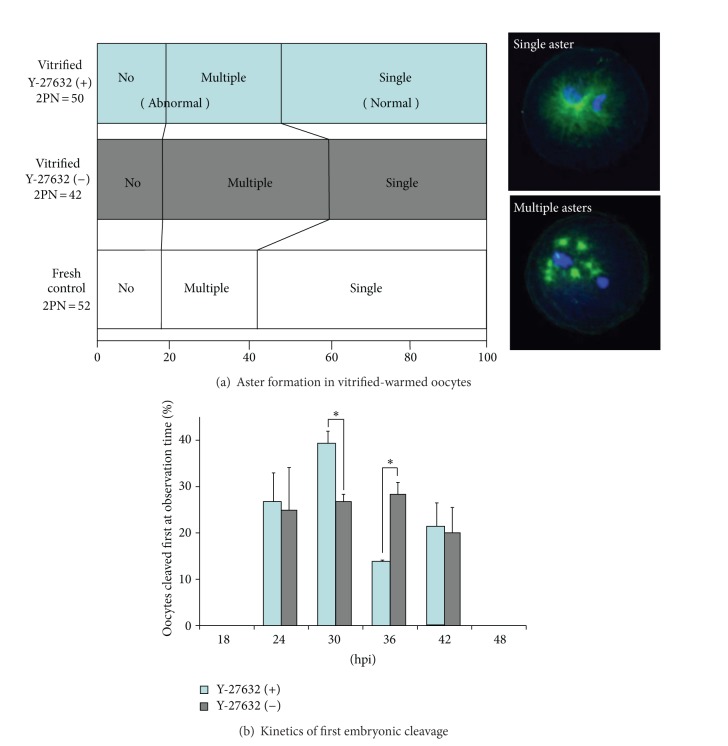
Effect of ROCK inhibition during postwarm recovery culture on revivability of bovine mature oocytes [85]. (a) Proportion of vitrified-warmed bovine oocytes exhibiting the formation of no, single, or multiple sperm aster(s). The abnormal incidence of multiple aster formation was inhibited by the recovery culture with Y-27632. Immunostaining against *α*-tubulin (green) and nuclear staining with DAPI (blue) were performed at 10-hour post-insemination (hpi). (b) Accelerated timing of first cleavage in bovine oocytes vitrified-warmed and rescued with Y-27632. Asterisks indicate significant difference at *P* < 0.05.

Therefore, we used the ROCK inhibitor (Y-27632; Figure 2(b)) to improve the developmental competence of vitrified-warmed oocytes during 2 hours of recovery culture after Cryotop vitrification [85]. The vitrification solution consisted of 15% EG, 15% DMSO, and 0.5 M sucrose, and oocytes retrieved from 1-day-stored ovaries (10–12°C) were subjected to the IVM. As summarized in Table 3, treatment of the postwarm mature oocytes with 10 *μ*M Y-27632 resulted in significantly higher oocyte survival rate prior to the IVF, Day-2 cleavage rate, and Day-8 blastocyst yield (21% versus 14%, fresh control; 34%). The resultant blastocysts in Y-27632-treated group had better quality in terms of total cell number and apoptotic cell ratio. Time-dependent change in mitochondrial activity of the vitrified-warmed oocytes was not influenced by ROCK inhibition during the period of recovery culture. However, the ability of ooplasm to support single-aster formation was improved by the ROCK inhibition (Figure 3(a)). Timing of first cleavage in the bovine oocytes vitrified-warmed and treated with Y-27632 was accelerated (Figure 3(b)), which may be favourable because bovine oocytes cleaving earlier are more likely to become blastocysts [110, 111]. Thus, inhibition of ROCK activity in vitrified-warmed bovine oocytes during short-term recovery culture could lead to higher developmental competence, probably due to decreased apoptosis and normalized function of the MTOC.

Using the same strategy, effect of two antioxidants, 10 *μ*M *α*-tocopherol (Figure 2(c)) or 250 *μ*M ascorbic acid, on rescuing vitrified-warmed bovine oocytes has been investigated in our laboratory. Oxidative stress by ROS must be one of the causes which may induce lipid peroxidation and/or organelle damage in bovine oocytes [112]. Interestingly, the supplementation of *α*-tocopherol, not ascorbic acid, to the recovery culture medium resulted in a significantly higher blastocyst yield from the postwarm oocytes as 37% versus 26% in the postwarm control oocytes (fresh control; 53%) (unpublished data of I. Yashiro and S. Hochi). The improved baseline of blastocyst yield in the nonvitrified control group was due to the availability of the fresh (=within 6 h after slaughter) bovine ovaries for recent experiments.

## 5. Conclusion

Ultrarapid vitrification procedure, originally reported using electron microscope grid as cryodevice [47], has become a standard approach for cryopreservation of bovine oocytes with some modifications. Due to numerous efforts, as the development of novel cryodevice such as OPS [48] or Cryotop [69] and the preloading with low concentration permeable CPA [49, 70, 113], blastocyst yields at >10% have been commonly reported by several laboratories during the last decade. Two recent attempts to improve cryosurvival of bovine oocytes have been focused on; the qualitative improvement of IVM oocytes prior to the vitrification and the short-term recovery culture of vitrified-warmed oocytes prior to the subsequent IVF. Supplementation of L-carnitine to the IVM medium of bovine oocytes has been reported to redistribute cytoplasmic lipid droplets and improve the cryotolerance of the oocytes after Cryotop vitrification as the blastocyst yield of 34% (comparable to fresh control) [84]. However, it is still unclear whether the positive effect of L-carnitine is reproducible. Incidence of multiple aster formation, a possible cause for low developmental potential of vitrified-warmed bovine oocytes [35], can be inhibited by a short-term culture of the postwarm oocytes in the presence of ROCK inhibitor, with a blastocyst yield of 21% after the Cryotop vitrification (>10% less than fresh control) [85]. Use of an antioxidant *α*-tocopherol during the recovery culture also rescued the postwarm bovine oocytes as the maximum blastocyst yield at 37% (>10% less than fresh control). Thus, chemical treatment of bovine oocytes before or after the vitrification protocol made it possible to increase their revivability to 20–40% when evaluated with blastocyst yield. Further improvements of the vitrification procedure, combined with pre- and postvitrification chemical treatment, would overcome the high sensitivity of bovine oocytes to cryopreservation and provide valuable information for biomedical experts working in human infertility clinic.

## Figures and Tables

**Table 1:** A list on Day-2 cleavage and Day-8 blastocyst yield from bovine mature oocytes cryopreserved and fertilized in vitro.

Years	Method/device for cryopreservation	Oocyte cryosurvival		Literature
		Cleavage rate	Blastocyst yield	
1992	Freezing/French straw	42%	3%	Otoi et al. [45]
1992	Vitrification/French straw	22%	9%	Hamano et al. [46]
1996	Vitrification/EM-grid^∗1^	40%	15%	Martino et al. [47]
1998	Vitrification/OPS^∗2^	50%	13%	Vajta et al. [48]
2000	Vitrification/Microdrop	62%	11%	Dinnyes et al. [49]
2004	Vitrification/Cryotop	70%	7%	Chian et al. [50]
2005	Vitrification/GL-tip^∗3^	49%	17%	Tominaga et al. [51]
2010	Vitrification/Cryotop	76%	12%	Zhou et al. [52]

^∗1^EM-grid: electron microscope grid; ^∗2^OPS: open-pulled straw; ^∗3^GL-tip: gel-loading tip.

**Table 2:** Supplementation effect of L-carnitine to IVM medium on the cryotolerance of bovine oocytes [84, 86].

L-Carnitine	Vitrification	Nuclear maturation	Survival	Cleavage	Blastocyst yield
Chankitisakul et al. [84]					
−	−	67%	92%^a^	84%^a^	44%^a^
+	−	65%	93%^a^	84%^a^	45%^a^
−	+		81%^b^	57%^b^	20%^b^
+	+		83%^b^	63%^b^	34%^a^

Phongnimitr et al. [86]					
−	−	68%^d^	100%	78%^c^	24%^d^
+	−	78%^c^	100%	76%^c^	31%^c^
−	+		86%	67%^d^	11%^e^
+	+		88%	69%^d^	13%^e^

Superscripts a versus b, c versus d versus e in each column: *P* < 0.05.

Concentration of L-carnitine in the IVM medium: 0.6 mg/mL.

**Table 3:** Rescue of vitrified-warmed bovine oocytes with ROCK inhibitor (Y-27632) [85].

Vitrification	Y-27632	Survival	Cleavage	Blastocysts		
				Yield	Cell number	Apoptotic cell ratio
−	−	100%^a^	71%^a^	34%^a^	135.7^a^	1.8%^a^
+	−	90%^b^	56%^b^	14%^c^	97.5^b^	4.0%^b^
+	+	98%^a^	72%^a^	21%^b^	124.6^a^	2.2%^a^

Superscript a versus b versus c in each column: *P* < 0.05.

Concentration of Y-27632 in recovery culture medium: 10 µM.
